# Learning Motivational Interviewing in VR: A Feasibility Study Using Self-Reported Measures

**DOI:** 10.1177/29941520251380697

**Published:** 2025-11-24

**Authors:** Daan J. Verhoeven, Floris T.J. Ferenschild, Bas Henk Verhoeven, Edward C. Bosma, Otmar Buyne, Harry van Goor

**Affiliations:** 1 Department of Surgery, Radboud University Medical Center, Nijmegen, The Netherlands.; 2 The Simulation Crew, Nijmegen, The Netherlands.; 3 E-infuse Interactive Online Postgraduate Course, Heilig Landstichting, The Netherlands.

**Keywords:** motivational interviewing, virtual reality simulation, medical students, communication skills training, usability evaluation, virtual patients

## Abstract

Competency in motivational interviewing (MI) for addressing lifestyle-related health issues is increasingly important for health care professionals, yet current simulation training is labor-intensive and expensive. Immersive virtual reality (VR) training may offer a cost-effective alternative. We developed a VR MI training, featuring animated virtual patients with common “lifestyle” issues (osteoarthritis, weight loss, smoking cessation) and evaluated its feasibility among medical students. The training consisted of two modules (MI basic techniques and MI communication processes). Each module included three scenarios (osteoarthritis, weight loss, smoking cessation). Both modules had to be completed twice within 1 week. Twenty fourth-year medical students (12 female, 8 male) completed two training modules twice at home using the Oculus Quest headset. Usability, learning motivation, VR immersion, discomfort, ability for exercising, learning, and proficiency in MI, were assessed with validated and customized questionnaires. Mean and standard deviation (SD) total exercise time was 99 and 29 min, respectively. Internal consistency of the questionnaires varied between questionable (alpha 0.650, VR immersion) and good (alpha 0.853; attention, relevance, confidence, and satisfaction model, motivation of learning). Mean system usability score was 69.5, with highest agreement on user-friendliness (>80%) and the lowest on frequency of use (30%) and confidence in using the VR application (36%). Only half of participants reported added value for their future profession. Ninety percent reported “realization being in a virtual environment”; 40% had empathy with the virtual patient. Outcomes of customized questions supported a well-designed, easy-to-use, time-balanced training tool, increasing self-confidence in motivational interviewing. Speech recognition issues causing unnatural conversational flow were reported as main drawback. VR-based MI training seems feasible and acceptable for unsupervised student use. Proficiency findings vary and should be interpreted with caution. Added value to skills acquisition and professional development is questioned, probably due to limitations in natural conversation. The small homogenous study group limits generalizability.

## Introduction

Motivational interviewing (MI) is a patient-centered communication method that supports patients in addressing unhealthy behaviors by enhancing their autonomy and exploring ambivalence about change.^1^ With the rising prevalence of lifestyle-related diseases, MI has become increasingly relevant in health care, offering a structured yet emphatic approach to promote behavioral change.^2^ MI is well aligned with modern patient-centered care principles and has demonstrated effectiveness in weight loss, smoking cessation, diabetes management, and medication adherence.^3–6^ Despite its proven value, motivational interviewing remains inconsistently embedded within medical curricula, and many physicians lack sufficient expertise to incorporate MI effectively in their practice.^7^ Learning MI is widely regarded as difficult to master due to the reflective nature of this conversation.^8,9^ MI training curricula lack standardization in structure, delivery, and assessment.^10^

MI is commonly taught through lectures, role plays, didactic exercises, demonstrations, and occasionally simulated patients.^10,11^ Training with simulated patients is known to replicate real clinical scenarios effectively, which students consistently value positively.^12–15^ However, simulated patient training poses financial and organizational challenges, with unclear cost-effectiveness.^13,16^

To address these limitations, virtual reality (VR) has emerged as a potential alternative to traditional teaching methods, potentially overcoming key barriers associated with actor-based training.^16–18^ VR facilitates repetitive practice, skill refinement, and personalized learning in a secure, low-pressure environment.^17–19^ VR can reduce logistical and personnel burdens, thereby offering greater flexibility and lowering costs.^17^ However, VR-based MI training may be limited by reduced emotional engagement, fewer nonverbal cues, and less communicative variation compared to real-life interactions.^17,20,21^

Several studies have explored VR for communication skills training in medical and nursing students. It is perceived as an effective tool for communication training and the practice of nontechnical skills.^22–24^ In nurse education, VR-based communication training can be as effective as live simulation, with additional benefits in accessibility and student engagement.^25,26^ VR has also been shown to improve students’ empathic communication skills, a key component of motivational interviewing.^19,27,28^ Finally, it can promote behavioral change among clinicians, particularly where traditional methods fall short.^29^

Building on our experience with co-designing and validating VR simulations for therapeutic and low-literacy communication using emotionally animated virtual humans (www.thesimulationcrew.nl), we developed a VR motivational interviewing training as part of a broader initiative to integrate lifestyle education into medical, nursing, and postgraduate curricula.^30^ This study evaluates usability, initial proficiency, and self-efficacy among medical student volunteers, performing the VR training unsupervised at home.

## Methods

### Design of the VR MI simulation

A development team, comprising learning experience designers, 3D artists and animators, software developers (The Simulation Crew, Nijmegen, The Netherlands), educationalists and clinicians (Radboud University Medical Center, HAN University of Applied Science and E-infuse), commenced the design and validation of the software system in 2020. Existing literature and guidelines on motivational interviewing were utilized to define the functionalities of the system.^1^ Findings and recommendations from Birckhead et al.’s step-up research methodology, albeit in clinical VR, were incorporated to ensure adherence to established best research practices and maximize the potential impact of the study.^31^

The VR tool was developed in Dutch. The development process involved iterative cycles of content and technical development, mid-term evaluation, optimization, and final evaluation by the development team. Initial validation was performed from July to December 2021 including five medical students and four experienced medical doctors daily practicing motivational interviewing. Feedback identified issues, such as limited speech recognition, unnatural pauses and mismatched responses, that affected the natural flow of conversation, and technical glitches, unclear instructions and limited scenario variation. The development team addressed these issues through software modifications, including text instructions to circumvent unnatural conversation, and optimizing the user interface (see Supplementary Data). Continuous refinement through participant feedback enhanced the VR training program’s content, usability, and educational value to a level of studying feasibility in an educational setting with appropriate end users. Considering the specific advantage of VR for place independent self-managed repetitive training, we choose the student home setting for this study.

### VR application

**FIG. 1.**
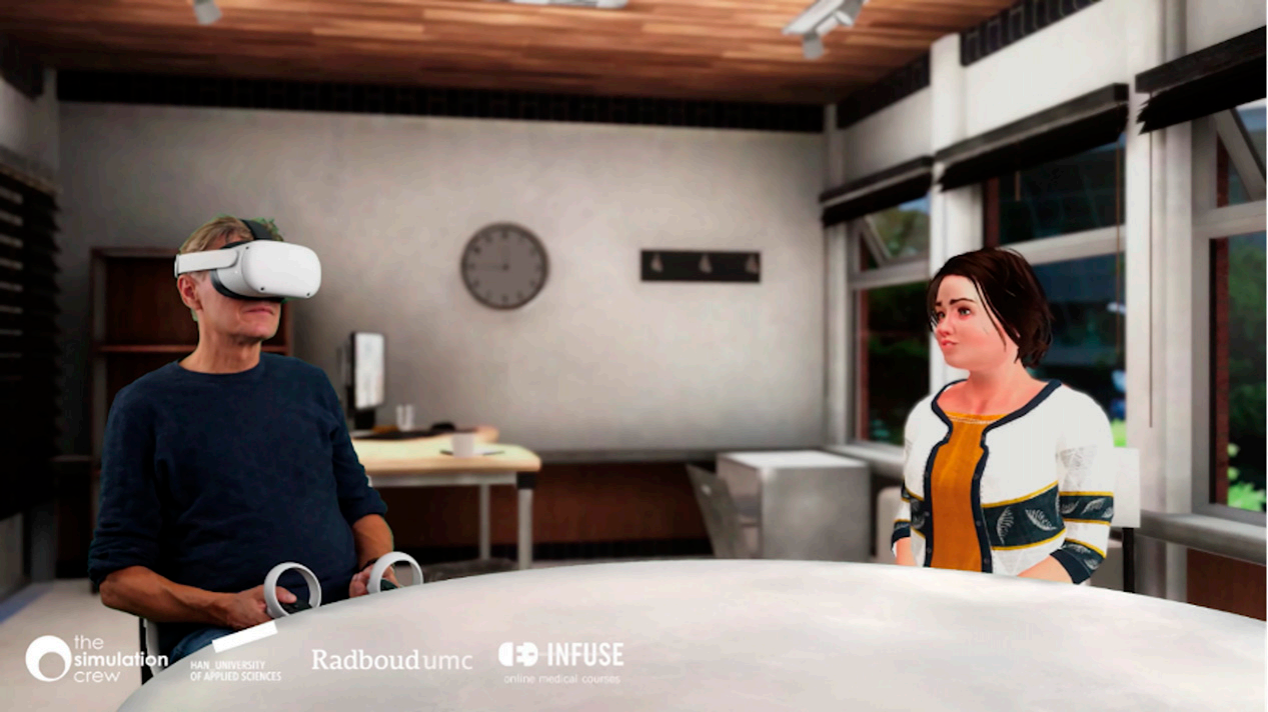
Composed picture of real and virtual environment; left trainee, right human avatar.

**FIG. 2.**
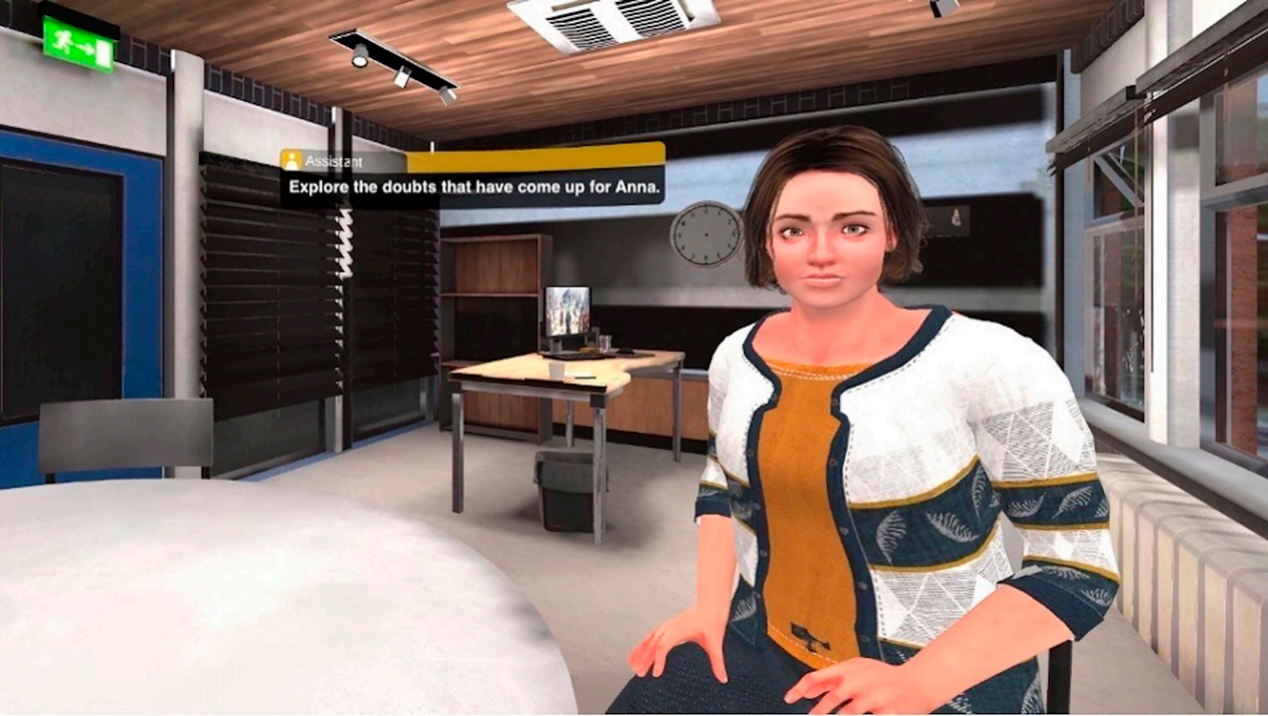
During the exercise, a general task description (Assistant) helps the user to conduct the conversation.

**FIG. 3.**
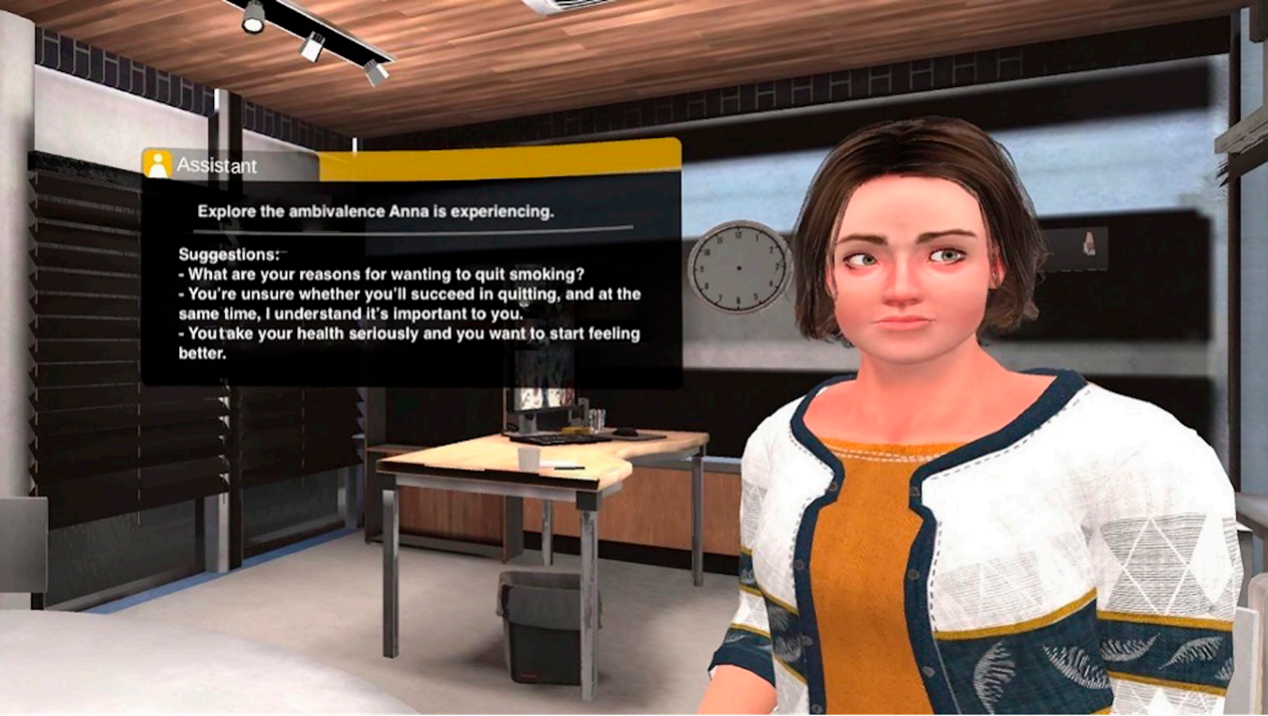
When a user is in need of more help, a context-specific help screen (Assistant) can be activated containing specific tips.

**FIG. 4.**
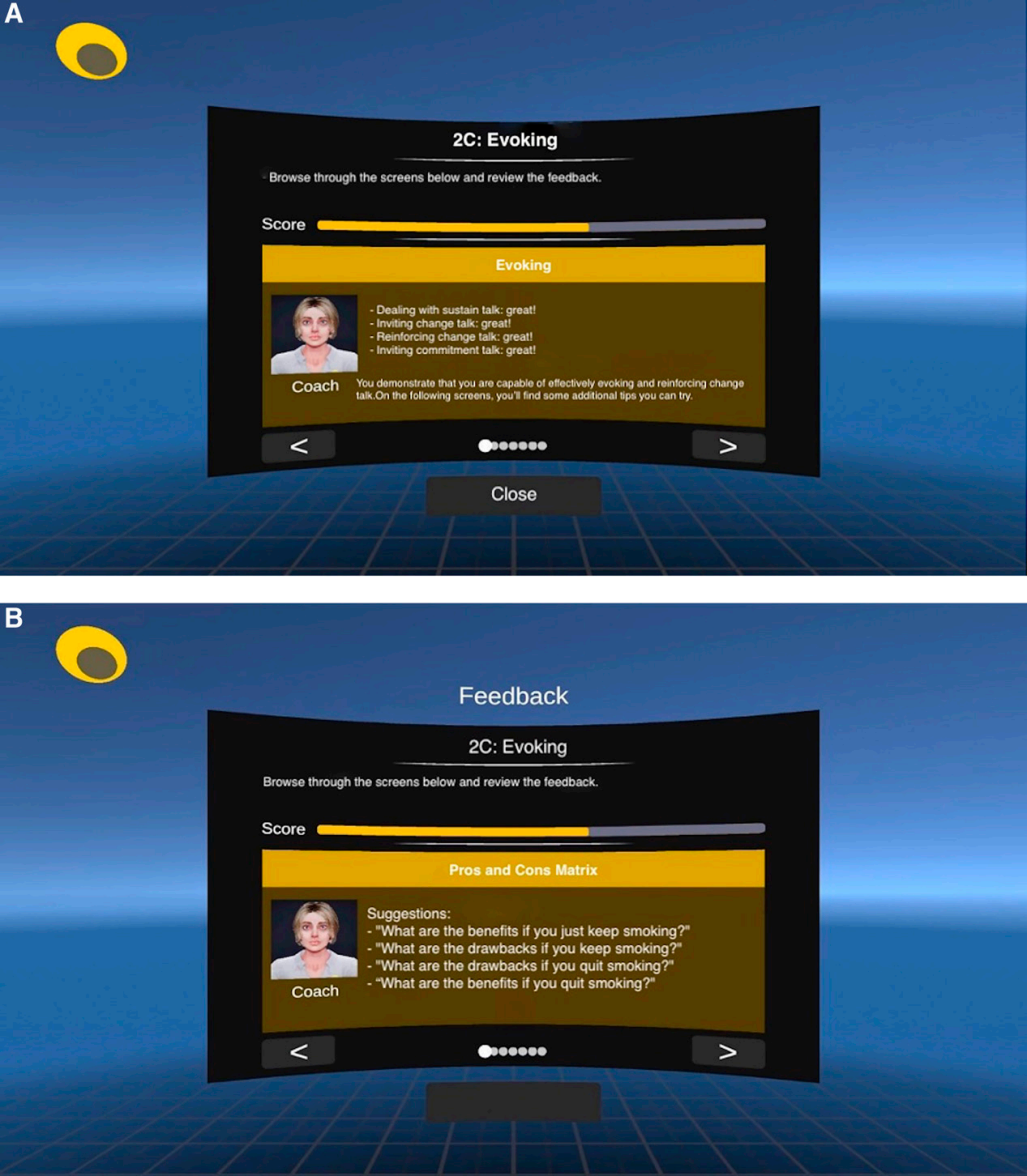
Upon completion of the exercises, automated feedback is generated and shown. **(A)** Overall score with explanation topic “evoking.” **(B)** Tip and suggestions use of advantage/disadvantage matrix to evoke discussion topics.

The MI simulation training consists of two modules: The Module Basic Techniques covers asking open questions, reflective listening, confirming, and summarizing. The Module MI Processes covers exercises in connecting, focusing, evoking, and planning. The training is based on the theoretical model of motivational interviewing as developed by Miller and Rollnick and as advocated by the Motivational Interviewing Network of Trainers (MINT) international organization (www.motivationalinterviewing.org).^1,32^ Each module consists of three simulation scenarios. The scenarios osteoarthritis, weight loss, and smoking cessation were chosen in agreement with clinicians, aiming at the most common clinical encounters where lifestyle is addressed. At the start of the training, participants enter a virtual medical office where they interact with a human avatar (Fig. 1). During the exercise, a general task description helps the user to conduct the conversation (Fig. 2). When a user needs more help, a context-specific help screen can be activated containing specific tips (Fig. 3). Upon completion of the exercises, automated feedback is generated and shown (Fig. 4a and 4b).

### Participants

Participants were fourth-year medical students (six-year program: 3 years bachelor, 3 years master internships) of the Radboud University Medical Center, who prepared for their 8-week surgical internship. All participants signed an informed consent form for voluntary participation and the use and storage of their data in a research database. No ethical review board approval was sought because this is not mandatory by Dutch law for this type of research.^33^ Students were explicitly informed that choosing not to participate would not have any adverse effects on their grades or academic progress. Students were not reimbursed for participation or received credit points.

### VR device

The head-mounted device (HMD) Oculus Quest was used (Meta Technologies Ltd., Menlo Park, CA, USA) with one-hand controller. The Oculus Quest is a wireless HMD with integrated speakers, microphone, and motion tracking, allowing participants to hear, see, and speak while interacting with the virtual environment. The HMD is connected to a Wi-Fi network to permit natural language processing. Virtual patients respond in real time using speech recognition-based algorithms that trigger pre-programmed verbal and nonverbal replies (see Supplementary Data). VR HMDs for this study were provided by The Simulation Crew free of charge.

### Simulation training setting and procedure

Participants had an individual intake at the Radboud University Medical Center explaining the study and the characteristics of the VR exercises. They were shown how to operate the HMD including how to properly use the headsets, connect to their home Wi-Fi network, troubleshoot technical issues, and contact for assistance. They were familiarized with the controller, particularly how to select the proper exercise, how to start and stop the audio streaming for the speech service, and how to activate the “tips.”

Each module had to be completed twice at home in 1 week, where the feedback after the first completion was to be used for the second completion. Participants were instructed to chart the exercises including any challenges, insights, or (second person) feedback they encountered while exercising at home. At the end of the week, participants returned the HMD device and completed the questionnaires.

### Measures and outcomes

Baseline data were collected regarding age, gender, self-reported proficiency level in motivational interviewing (beginner, advanced, competent) and self-reported exercise time. Exercise time was the time used to complete two sessions. A session was defined as completion of both modules and all three scenarios in each module. A combination of previously validated questionnaires along with specific questions related to the simulation training was used for measuring usability, motivation of learning, VR immersion, the applicability of the virtual environment, the ability for exercising and learning proficiency in motivational interviewing, and discomfort using the VR HMD. Usability was measured with the system usability scale (SUS, ranging from 0 [none] to 100 [maximal]), motivation of learning with the Keller’s ARCS (attention, relevance, confidence, and satisfaction) model, immersion with the VR immersion questionnaire, proficiency through self-assessment using the Dreyfus model, and discomfort with the Misery Scale (ranging from 0 [no discomfort] to 10 [vomiting]).^34^ The set of specific questions regarding the software training application and use was composed by members of the development group and tested for clarity and consistency by educationalists experienced in qualitative research. This set also contains seven questions (out of 25) related to immersion in the conversation and interaction with the virtual patient.

### Data and statistical analysis

Considering the explorative and qualitative nature of the study, we did not perform a power analysis for number of participants. Demographics and outcomes of questionnaires were descriptively analyzed. Internal consistency of the usability, motivation, and VR immersion questionnaires and the set of specific questions was evaluated using Cronbach’s alpha test statistic. A value >0.6 is considered questionable, >0.7 acceptable, >0.8 good, and >0.9 excellent.^35^

The Statistical Package for Social Sciences version 27.0 (SPSS, IBM corp.) was used. Graphs were generated using Microsoft Excel version 2208 (Excel, Microsoft corp.).

## Results

### Demographics

Twenty students—12 females and 8 males—with an average age of 24.1 years with a standard deviation (SD) of 1.9 years, participated in the study. All students completed both modules twice. Mean total exercise time was 99 min with an SD of 29 min. Ten participants considered themselves beginners, five advanced beginners, and five competent in motivational interviewing (Dreyfus self-assessment tool). Three students reported a score of 1 on the Misery Scale, indicating some discomfort using the HMD, but no specific symptoms.

### Usability (SUS)

**FIG. 5.**
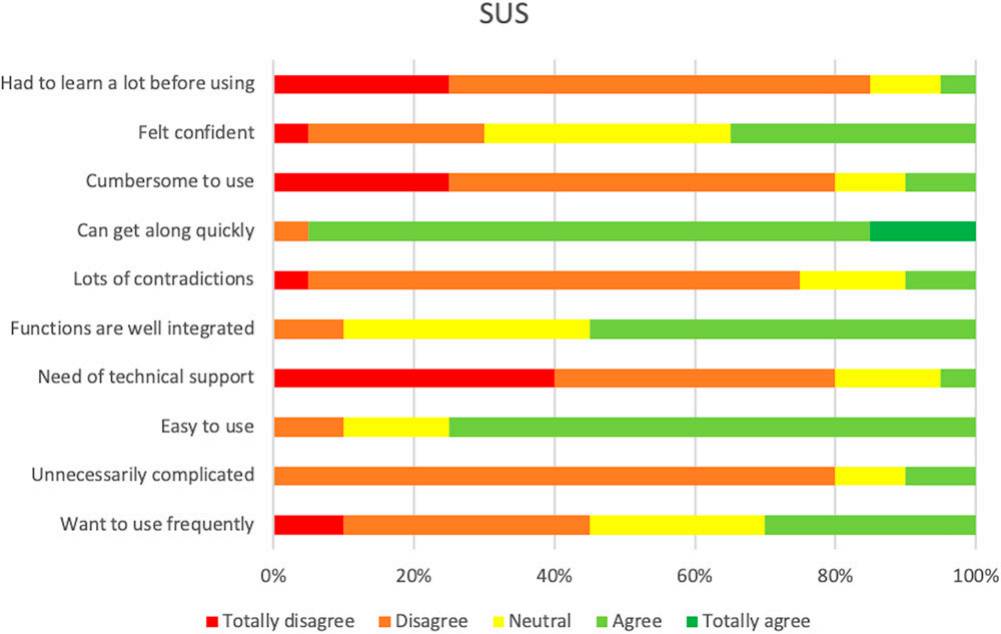
Percentage of participants per category of the system usability scale (SUS) agreement and individual statement; 100% is 20 participants.

Students scored overall usability of the VR training with a mean of 69.5 (see Fig. 5). Cronbach’s alpha coefficient was 0.723, indicating acceptable consistency. Participants were positive about the user-friendliness of the VR application with 95% (totally) agreeing with the statement “being able to get along quickly” and 84% (totally) disagreeing with the statement “I had to learn a lot before I could use it.” Fifty-four percent did not want to use the VR application frequently, with another 26% being neutral.

### Motivation (ARCS)

**FIG. 6.**
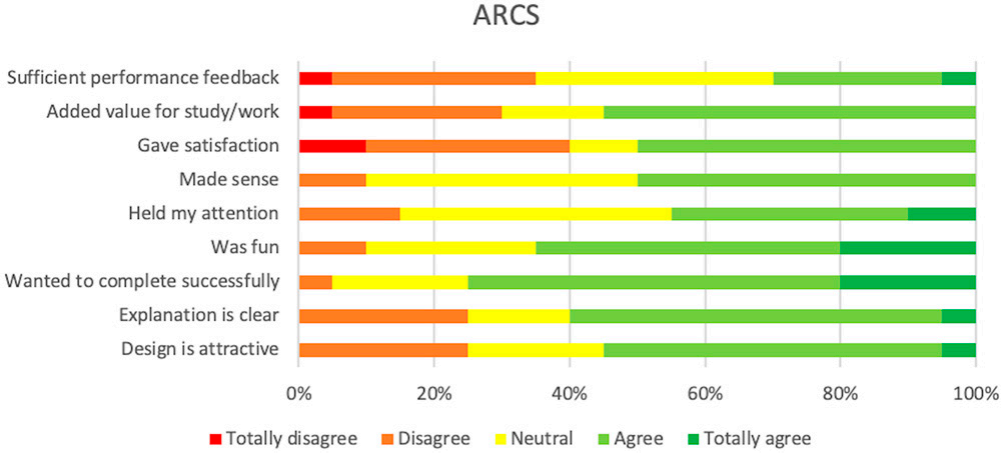
Percentage of participants per category of ARCS (attention, relevance, confidence, and satisfaction) model, motivation of learning and individual statement; 100% is 20 participants.

Cronbach’s alpha coefficient was 0.853, indicating good consistency. Over 50% participants (totally) agreed with statements regarding completion of the exercises, attractiveness of the design, and satisfaction to perform (see Fig. 6). Thirty percent valued the training adding to their education or work, with 35% being neutral and a similar percentage (totally) disagreeing.

### Immersion VR

**FIG. 7.**
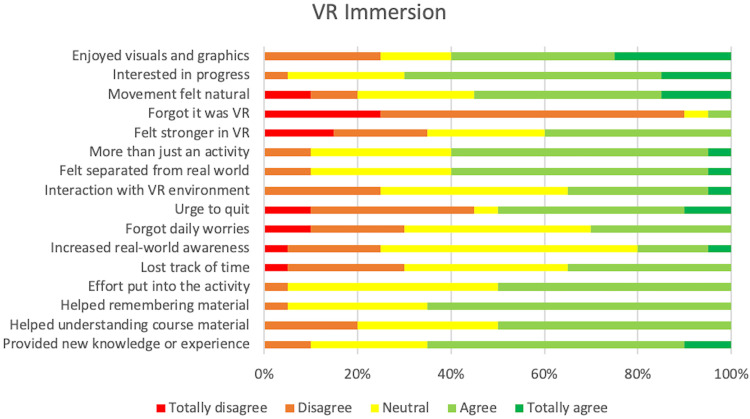
Percentage of participants per individual statement of VR immersion; 100% is 20 participants.

There was questionable consistency in answers on the VR immersive items with a Cronbach’s alpha coefficient of 0.650. Highest percentages of (total) agreements (>60% of participants) regarded the questions “enjoyed the visuals and graphics,” “interested in the progress,” “helped with remembering the material,” and “new knowledge of new experiences” (see Fig. 7). Ninety percent of participants totally disagreed with the statement “so involved that you didn’t realize it was VR.”

### Specific questions about the software training application and use

**FIG. 8.**
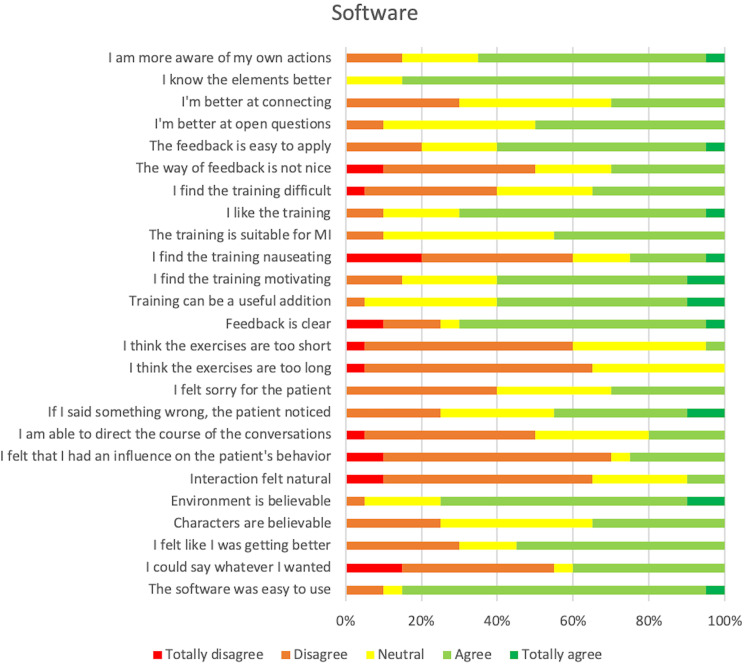
Percentage of participants per individual statement of software application and use; 100% is 20 participants.

Participants’ answers are shown in Figure 8. Cronbach’s alpha coefficient was 0.704, indicating acceptable consistency. Over 60% of (total) agreements regarded the statements “Like the training,” “Feedback is clear,” “Environment is believable,” and “Software was easy to use.” Statements concerning the interaction with the virtual patient, e.g., ability to direct the conversation, influence the patient’s behavior, and a feeling of a natural interaction scored low in agreement. Still, 30–45% of participants showed empathy and engagement with the virtual patient (“I felt sorry for the patient,” “If I said something wrong, the patient noticed”). Exercise duration was considered good, reflected by the majority disagreeing with either the statement that the exercise was too long or that the exercise was too short. Forty-five percent of students found the simulation suitable for training motivational interviewing with another 45% being neutral and only 10% with a negative opinion. Fifty-five percent felt they had improved in motivational interviewing.

## Discussion

The purpose of this feasibility study was to obtain user experiences of medical students in their clerkship phase, using a new self-administered VR motivational interviewing simulation training application for counseling virtual patients, that express common behavioral health issues like smoking, obesity, and physical inactivity. User experiences were collected through a variety of questionnaires addressing usability, applicability, acceptability, potential learning value, and VR immersion, presence, congruence, and plausibility. Results point in the direction of a promising tool to train behavioral counseling and improve learning outcomes for students in their clerkship phase, with added value to current skills training. However, findings were conflicting regarding perceived added value for professional practice. Results should be interpreted with caution considering generalizability due to the limited homogeneous sample. There appeared to be a substantial interindividual variation in VR immersion and plausibility that affected the relational components of the counseling exercises. However, interpretation of immersion-related findings should be cautious due to questionable internal consistency.

The favorable system usability outcome scores likely benefitted from the prior thorough iterative co-design process with similar end users. Almost all participants found the application easy to use. This is an important finding against the background of self-directed use at home without immediate technical and operational support. The self-directed use at home acknowledges the strength and contributed value of VR as a repetitive and spaced learning tool. However, this approach introduces risk of noncompliance and absence of immediate correcting feedback by faculty. In this software version we incorporated buttons with written information to assist learners in continuing the counseling, which was appreciated but at the same time noted as an artificial break of the conversation with the virtual patients.

All students reported completion of both modules twice, showing overall compliance. However, exercise time of about 50 min per completion of two modules with a substantial individual variation, might raise questions about the appropriateness of the exercise duration for each scenario and for each participant. Unfortunately, we could not track user data from the HMD to analyze exercise time in more detail. Only one-third expressed a desire to use the application more frequently. Participants may have misinterpreted this question in the SUS questionnaire, considering the frequency of use of the VR application for completing the assignment within 1 week. Alternatively, the extracurricular and research setting and the unfamiliarity with VR skills training, combined with the positive experience of training consultation skills with simulated patients or actors in the first 3 years of medical school, have contributed to this negative outcome. Further, the limited “richness” in the conversations of this VR application may have affected this result.

Three users experienced short-lasting nausea during home exercises. Clues why this occurred were not asked for in the questionnaire. Although this number is consistent with previous findings on the prevalence of VR sickness, it may highlight a potential drawback of unsupervised use at home, where no possibility of immediate corrective actions can be taken if symptoms arise.^36,37^

About half of the students did not perceive added value of the VR training for their future profession. This contrasts with the clear relevance of motivational interviewing in clinical practice.^9,38,39^ A lower perceived added value can be due to unawareness of the place of MI in their future profession. Many students have not decided yet about their future career in the beginning of their clerkship phase. Findings from the CHOosing clerkships In mediCal Education (CHOICE) study indicate that the clerkship years contribute to the students’ specialty choice.^40,41^

Besides a general questionnaire on VR immersion, we included specific questions on the plausibility and realism of the virtual patients and the interaction. Although half of participants felt that the virtual patient noticed a “wrong” question and one-third expressed an emotional connection “feeling sorry for the patient’, most did not experience the interaction during the conversation as real. This was predominantly due to an inability of the virtual patient to adequately address a question or comment leading to a break in the conversation and simultaneously a break in virtual presence of the student. This may have been emphasized by an absence or incomprehensible body language of the virtual character. A conversational break with learner’s discomfort and rethinking how to continue the consultation is normal in training motivational interviewing regardless of the simulation method and use of real or virtual humans. Real volunteer patients or actors, however, can assist in continuing a natural conversation in various ways unlike the virtual patients in this application. This drawback was even more apparent in piloting this version in a group of medical specialist trainees, having completed an online theoretical course on lifestyle intervention. Although the trainees recognized the value of practicing motivational interviewing in VR, the conversational flow did not align with their real-life counseling experiences in clinical practice (data not shown).

VR motivational interviewing skills training for improving lifestyle in medical education is new. The most common communication training scenarios in medical schools using virtual patients are history taking and bringing bad news. These are typically screen-based applications presented in a first-person perspective and incorporate (textual) instructions, tutorials, and feedback.^42^ Of the eight comparative studies in this systematic review on virtual patient simulators for medical communication training, four reported significant improvements in communication attitudes or skills following a virtual patient intervention.^42^ Effectiveness appeared to depend on pre- and post-training activities (instructive tutorial, debrief, reflection) and on human feedback, rather than on feedback provided by the VR system, which was part of our intervention.

Most VR research on motivational interviewing focuses on training general or specialist practitioners in behavioral counseling skills, typically within educational course or curriculum.^43,44^ A mixed-methods study among 14 pediatric residents, who completed a behavioral health anticipatory guidance VR curriculum, found high levels of immersion, spatial presence, and cognitive involvement, as well as increased perceived knowledge and skills in motivational interviewing.^45^ An effectiveness study among 55 residents demonstrated improved behavioral health anticipatory guidance and motivational adherence behaviors ∼8 weeks after a VR intervention compared to a control group.^46^ Higher motivational interviewing skill scores were observed in participants who trained with a virtual (computerized) standardized patient compared to those who studied a summary document from a computer MI training course by experienced health care professionals employed in a Veteran Administration Hospital.^44^ Sustained improvement at 3 months was observed on the “reflection-to-question ratio” subscale following repeated VR training. Although not thoroughly studied, most papers point out the ability of VR as an effective, safe, feasible, and scalable educational method for teaching evidence-based communication skills, like motivational interviewing, in settings that resemble the clinical setting.

### Strengths and limitations

A strength of this study is the co-created, well-designed, and evidence-based VR educational intervention, including a variety of virtual patients with different common lifestyle issues, intended to serve as a self-managed and “system-supervised” education tool for medical students to become skilled in motivational interviewing. However, the choice of piloting the intervention for usability in an unsupervised setting may have limited the interpretation of a few results, particularly those regarding motivation, session duration, and completeness of individual training sessions. Using self-reported measures through questionnaires is susceptible to bias, e.g., memory, social desirability and response bias, and measurement errors. However, in the phase of evaluating usability and acceptability of a VR application, self-reported outcomes can provide valuable insight and may be preferred over expert observation or quantitative measures.^31^ Skills proficiency outcomes should be interpreted with caution due to overestimation by self-reporting. Performance and improvement in skills are more accurately assessed by expert-rated measures.^42^ The same applies to the evaluation of the effect of the system feedback on performance in the repeated session. Conducting the study outside the standard communication skills curriculum, without preparatory instruction or simulated patients, may have affected students’ perceptions of the place of VR in mastering motivational interviewing.^47^

We used pre-programmed algorithms based on natural language processing that support structured teaching and learning motivational interview. However, these algorithms limit the natural flow and richness of the conversation. Current developments using different AI technologies for detecting multiple intents and recognizing nuances in users’ speech input for generating appropriate responses presented by real-time voice and animation of the virtual patient are promising and can solve current shortcomings in vividness of the conversation. Nevertheless, large language models introduce new challenges, including potential latency in responses and the risk of generating inappropriate or unrealistic replies, particularly in a complex conversation skills training.

The participating fourth-year medical students had initial experience with medical communication styles related to disease management but were generally inexperienced in motivational interviewing for lifestyle changes. The sample from one institution is homogeneous; the size is limited. This is acceptable for a feasibility study but limits generalizability. For students in curricula with an established lifestyle education program and for experienced health care workers with clinical experience, this VR basic MI skills training will have limited added value.^9,11^

The present study is part of a series of (research) initiatives aimed to transform medical communication skills training through VR. This transformation is necessary for several reasons. First, it may offer students the opportunity to train multiple times over an extended period of time, independent of place and time, and with easy access for low cost.^43^ In contrast, current training methods such as in-person role-playing and face-to-face standardized patient interactions, are typically one-off experiences and require extensive logistical planning and expensive infrastructural and human resources. Second, VR allows for the inclusion of a wide variety of behavioral counseling topics and diverse patient characteristics, which can be tailored to the trainee’s level of experience. Just-in-time exposure to a range of complex, lifelike scenarios may enhance competency and confidence in medical communication. Third, VR offers the opportunity to assess the influence of learner-related factors on performance by analyzing data derived through HMDs, whether or not in combination with wearable device data. Insights into emotional stress, focused attention, and situational awareness, for example, via eye tracking, have become a topic of growing interest in simulation-based skills training, particularly for providing contextual feedback, supporting adaptive learning, and individualized teaching plans.^48,49^

Considering the outcomes of the present study, the next version of the VR motivational interviewing training will be designed with the integration of current artificial intelligence speech technology. Simultaneously, virtual characters, behavioral counseling topics, and immersive environments will expand and adapt to suit a broader range of health and social care professionals (trainees).^9,11^ Additionally, the current version is being implemented in a disease prevention teaching module at the University of Applied Sciences of Arnhem and Nijmegen and is being evaluated for its potential to prepare for, or possibly replace, a motivational interviewing role-play workshop. This type of practice-oriented educational research is essential to define the role and value of VR training within the continuum of teaching methods from “know to does.” It also helps to transform communication skills training into true competency-based education that better prepares students for engaging with all types of actual patients.^50^

## Conclusion

This study demonstrates the usability and acceptability of a VR application for self-directed, home-based training in motivational interviewing skills. Self-reported proficiency outcomes varied and should be interpreted with caution. Disrupted conversational flow due to speech recognition was considered a major drawback and seemed to affect perceived added value to skills training and performance in clinical practice. Next steps include developing the next-generation VR MI simulation training with computer automated assessment, conducting a controlled trial by comparing VR with real volunteer patients or actors, and integrating VR MI simulation training into the current behavioral counseling curriculum.

## Abbreviations Used

ARCS: attention, relevance, confidence, and satisfaction model
HMD: head-mounted device
MI: motivational interviewing
MINT: Motivational Interviewing Network of Trainers
SUS: system usability scale
VR: virtual reality
